# Investigating the Crystallization Process of Boron-Bearing Silicate-Phosphate Glasses by Thermal and Spectroscopic Methods

**DOI:** 10.3390/molecules27030867

**Published:** 2022-01-27

**Authors:** Magdalena Szumera, Barbara Łagowska, Justyna Sułowska, Piotr Jeleń, Zbigniew Olejniczak, Radosław Lach, Anna Berezicka, Agnieszka Kijo-Kleczkowska

**Affiliations:** 1 Faculty of Materials Science and Ceramics, AGH University of Science and Technology, A. Mickiewicza 30, 30-059 Krakow, Poland; lagowska@agh.edu.pl (B.Ł.); sulowska@agh.edu.pl (J.S.); pjelen@agh.edu.pl (P.J.); Radoslaw.Lach@agh.edu.pl (R.L.); berezicka@agh.edu.pl (A.B.); 2 The Henryk Niewodniczanski Institute of Nuclear Physics, Polish Academy of Science, Radzikowskiego 152, 31-342 Krakow, Poland; zbigniew.olejniczak@ifj.edu.pl; 3 Faculty of Mechanical Engineering and Computer Science, Czestochowa University of Technology, Armii Krajowej 21, 42-201 Czestochowa, Poland; a.kijo-kleczkowska@pcz.pl

**Keywords:** silicate–phosphate glasses, devitrificates, phase separation, crystallization, structure

## Abstract

Glasses and devitrificates from the SiO_2_–B_2_O_3_–P_2_O_5_–K_2_O–CaO–MgO system with constant contents of SiO_2_ and P_2_O_5_ network formers, modified by the addition of B_2_O_3_, were analyzed. All materials were synthesized by the traditional melt-quenching technique. The glass stability (GS) parameters (K_rg_, ∆T, K_W_, K_H_) were determined. The effect of the addition of B_2_O_3_ on the GS, liquation phenomenon, crystallization process, and the type of crystallizing phases were examined using SEM-EDS, DSC, XRD, and Raman spectroscopy imaging methods. It was observed that the addition of B_2_O_3_ increased the tendency of the glass to crystallize. Both phosphates (e.g., Ca_9_MgK(PO_4_)_7_, Mg_3_Ca_3_(PO_4_)_4_), and silicates (e.g., K_2_Mg_5_(Si_12_O_30_), CaMg(Si_2_O_6_), MgSiO_3_) crystallized in the studied system. The Raman spectrum for the orthophosphate Mg_3_Ca_3_(PO_4_)_4_ stanfieldite type was obtained. Boron ions were introduced into the structures of crystalline compounds at high crystallization temperatures. The type of crystallizing phases was found to be related to the phenomenon of liquation, and the order of their occurrence was dependent on the Gibbs free enthalpy.

## 1. Introduction

It is known that crystallization processes, such as the crystallization of a glass, consist of three fundamental steps: the attainment of metastability, the formation of nuclei, and crystal growth [1]. When a liquid is cooled below its melting point, crystal nucleation can occur homogeneously or heterogeneously in the body of the liquid or on the surface. A typical glass-forming silicate melt is unique among solutions due to its ability to dissolve nearly all chemical elements. Such a melt is, therefore, a solution of oxides present in a thermodynamic equilibrium. Another unusual property of a glass-forming melt is its enormous change in viscosity with temperature. The viscosity of the material increases with cooling, but at certain temperatures, the mobility of molecules may still be great enough for a crystalline phase to form [2].

Liquation is considered a precrystallization ordering process [3]. Glasslike regions may form with the composition of the future crystals without simultaneous crystallization of these regions. Therefore, crystallization is preceded by a certain independent effect of primary precrystallizational demixing accompanied by a gain in thermodynamic potential. Separation of the precrystallizational phase (demixing) occurs by way of forming critically sized amorphous nuclei that are capable of further growth [4]. Precrystallization microphase separation is analogous to the usual metastable phase separation of the liquid type, except that, first, in usual liquid metastable phase separation, the composition of the glasses deposited may differ greatly from the composition of the possible crystals and, second, it may appear and disappear reversibly without crystallization, depending on the temperature. Moreover, metastable phase separation occurs only in certain glasses in a definite composition region, whereas crystallization of glasses is possible with any composition and is irreversible below the solidus temperature. If metastable liquation were a purely precrystallization effect, it could not disappear with a rise in temperature once it had occurred, culminating in crystallization below the solidus [4]

The crystallization of multicomponent glasses consisting of two glass formers, i.e., SiO_2_ and P_2_O_5_, SiO_2_ and B_2_O_2_, or P_2_O_5_ and B_2_O_3_, is particularly complex. According to the literature, the crystallization process occurs in several stages, beginning with the formation of simple compounds built from chemical elements that are weakly bound to the glass structure and, therefore, are most mobile at a given temperature [5,6]. They play the role of nucleating agents. Compounds with more complex structures are formed in the next stages depending on the chemical composition of the glass.

In ternary system glasses, in which the matrix is formed by SiO_2_ and B_2_O_3_ and one modifying component, the crystallization process generally has a single-stage or multi-stage character. The products of crystallization are usually borates (β-BaB_2_O_4_ [7], Ba_2_Zn(BO_3_)_2_ [8], Mg_2_B_2_O_5_ [9], CaB_2_O_4_ [10]) and silicates (Ba_5_Si_8_O_21_ [7], BaZnSiO_4_ [8], CaSiO_3_, Ba_4_Si_6_O_16_ [10]) in lower and higher temperature ranges, respectively. In the case of glasses containing P_2_O_5_ and B_2_O_3_ as matrix formers, the crystallization has a multistage character and the main products, at both lower and higher temperatures, are phosphates, e.g., Zn_2_P_2_O_7_ [11], Li_3_Fe_2_(PO_4_)_3_, LiFeP_2_O_7_ [12], BaP_2_O_5_, BaBPO_5_, and BPO_4_ [11,13]. It is worth noting that the presence of boron ions in the structure of crystallizing phases is observed mainly at higher temperatures (above 590 °C) and at the minimum B_2_O_3_ content of 6 mol.% [11,13]. Lopes et al. [7] linked the type of crystallizing phase with the activation energy for crystallization. The authors of the publication concluded that the formation of the barium borate crystalline phase requires a lower activation energy, as it is generated in a loose borate-rich glass network structure, while the barium silicate crystalline phase has to be formed in a more interlocked silicate glass network structure. A similar approach to the study of glass crystallization was adopted by Kržmanc et al. [9], Liu [12], and Kalenda [13], while Zhu et al. [10] also considered the influence of the size of ions building the glass matrix on the type of crystallizing phase. Both Zhu et al. [10] and Hu et al. [14] agreed that the larger a cation’s size, the lower its mobility, and thus the smaller the possibility of its occurrence in the crystallizing phases.

It is known that B_2_O_3_ is a strong glass networker; its presence in minor amounts possibly decreases the number of bridging oxygen atoms in silicate-based networks; thus, it may decrease the viscosity of the glass. Such behavior, which increases the mobility, is expected to promote devitrification at lower temperatures. This feature was exploited by Gou et al. [15], who modified 45Si5S glass (Hench glass) with B_2_O_3_ (0–6 mol.%) to decrease the heat treatment temperature of glass–ceramic analogues. The research shows that B_2_O_3_, apart from significantly decreasing the heat treatment of B_2_O_3_ containing 45S5 analogous, causes the desired phase of Na_2_Ca_2_Si_3_O_9_ to crystallize.

Fabert at al. [16] studied the partial to full substitution of SiO_2_ with B_2_O_3_ in order to develop bioactive glasses with optimum thermal processing windows. Typical silicate bioactive glasses, such as S53P4 and 45S5, are known to be prone to crystallization upon heating. It has been shown that while borate glasses are unsuitable due to their limited hot forming domain, borosilicate glasses possess not only a wide hot forming domain but also a low activation energy for viscous flow. The crystallization rate of these borosilicate glasses was found to be lower than those of their borate and silicate counterparts.

In this work, we present a crystallization study of the multicomponent glasses from the SiO_2_–B_2_O_3_–P_2_O_5_–K_2_O–MgO–CaO system where the glass modifiers MgO and CaO are replaced in stages by B_2_O_3_. Such a study has not previously been reported in the literature. The studied materials have an interesting application as glassy fertilizers that introduce into the soil environment, among others, boron and important microelements [17,18]. However, detailed knowledge of their structure and stability is required to optimize their properties. For this reason, the effects of the B_2_O_3_ content and glass modifiers, such as MgO, CaO, and K_2_O, on the type of crystallizing phase was studied. The results of thermal tests were analyzed with structural studies (X-ray diffraction method) and microscopic observations (SEM-EDS analysis), including Raman spectroscopic imaging (RSI).

## 2. Materials and Methods

### 2.1. Synthesis of SiO_2_–B_2_O_3_–P_2_O_5_–K_2_O–CaO–MgO Glasses

Silicate–borate–phosphate glasses of SiO_2_–B_2_O_3_–P_2_O_5_–K_2_O–MgO–CaO composition were obtained by conventional melting of the respective chemically pure reagents (SiO_2_, (NH_4_)_2_HPO_4_, K_2_CO_3_, MgO, CaCO_3_, and H_3_BO_3_) in platinum crucibles in the 1400–1450 °C temperature range. Glasses were quenched by immersing the crucibles in water. The choice of chemical composition of the tested glasses was based on previous studies of silicate–phosphate glasses used for agricultural applications [18,19]. The SiO_2_ (41 mol.%), P_2_O_5_ (6 mol.%), and K_2_O (6 mol.%) contents were kept constant in all glasses, whereas the amount of B_2_O_3_ (2–28 mol.%) increased at the cost of MgO and CaO, with the MgO/CaO ratio kept constant. The chemical composition of examined glasses is presented in Table 1. The amorphous character of the samples was confirmed by the X-ray diffraction method, which indicated that a fully amorphous material could only be obtained when the content of B_2_O_3_ did not exceed 25 mol.% [19].

### 2.2. Characterization

The FEI Nova NanoSEM 200 scanning electron microscope was used for SEM-EDS examinations of both glasses and their devitrificates. The observations were carried out under high vacuum conditions with a back scatter electron detector (BSE) and an accelerated voltage equal to 18 kV.

The thermal behavior of samples was studied with the STA 449 F3 Jupiter (NETZSCH) operating in DSC mode. Fifty milligram samples were ground to a grain size of 0.1–0.3 mm and placed in open Pt crucibles. The DSC measurements were carried out at a heating rate of 10 °C min^−1^ under the flowing air atmosphere (40 mL min^−1^). Al_2_O_3_ was used as the reference material.

The glass stability (GS) [20] of studied glasses was determined by DSC analysis. The parameters describing the glass stability can be derived from the characteristic glass heating process temperatures T_g_, T_x_, T_c_, and T_m_, which correspond to the glass transition onset and the onset of and maximum crystallization peak, and the melting temperature, respectively. The most widely used are the Hruby [21], Weinberg [22], Lu and Liu [23], and Kosidis–Petrovic [20] parameters, which are defined by the following equations:

K_H_ = (T_x_ − T_g_)/(T_m_ − T_x_) (1)

K_W_ = (T_c_ − T_g_)/T_m_ (2)

K_rg_ = T_g_/T_m_. (3)

Since the difference between T_x_ (or T_c_) and T_g_ is another indicator of GS, it was also included in the analysis:

ΔT = T_x_ − T_g_ (4)

The DSC curves were evaluated with Proteus software (NETZSCH).

Once the crystallization temperatures of the selected samples were determined by DSC, the phase composition of the devitrificates was identified by XRD. For that purpose, the fraction containing the 0.1–0.3 mm particles was separated from the sample and heated isothermally for 12 h at the designated crystallization temperature. The phase compositions of the samples were identified via the X-ray diffraction analysis, based on the ICDD databases. XRD measurements were performed using a Empyrean diffractometer (Panalytical). The number of phases was determined through the Rietveld quantitative phase analysis using the High Score Plus software. The phase composition of the samples was quantified by Rietveld refinement and X-ray diffraction. X-ray diffraction patterns were obtained with a continuous scan. Empyrean (Malvern Panalytical) diffractometer fitted with a Cu tube operating at 45 kV, 30 mA was used to obtain data for the Rietveld refinement over the 5–90° 2θ with a step size of 0.08. The total measurement time was four hours. The content of the amorphous phase was found by applying the internal Al_2_O_3_ standard.

Raman imaging studies were carried out using a WITec Alpha 300 M+ spectrometer equipped with a 488 nm diode laser (laser spot was approx. 650 nm), 1800 grating, and a 100× ZEISS Epiplan Neofluar objective. Imaging was conducted in a 20 × 30 µm area and the scan parameters were as follows: 80 lines with 120 points per line and 120 lines with 80 points per line for the 2B and 20B samples, respectively. The single accumulation time was set to 1 s. All mathematical corrections were done using WITec Project Five 5.2 Plus software. In order to determine the chemical composition of the measured surface, a true component analysis was done. As a result, images as well as spectra of component distributions were obtained. The reference spectra were taken from the Horiba JY Raman Library (July 2008).

## 3. Results and Discussion

### 3.1. Characteristics of the Analyzed Glasses

**Figure 1:**
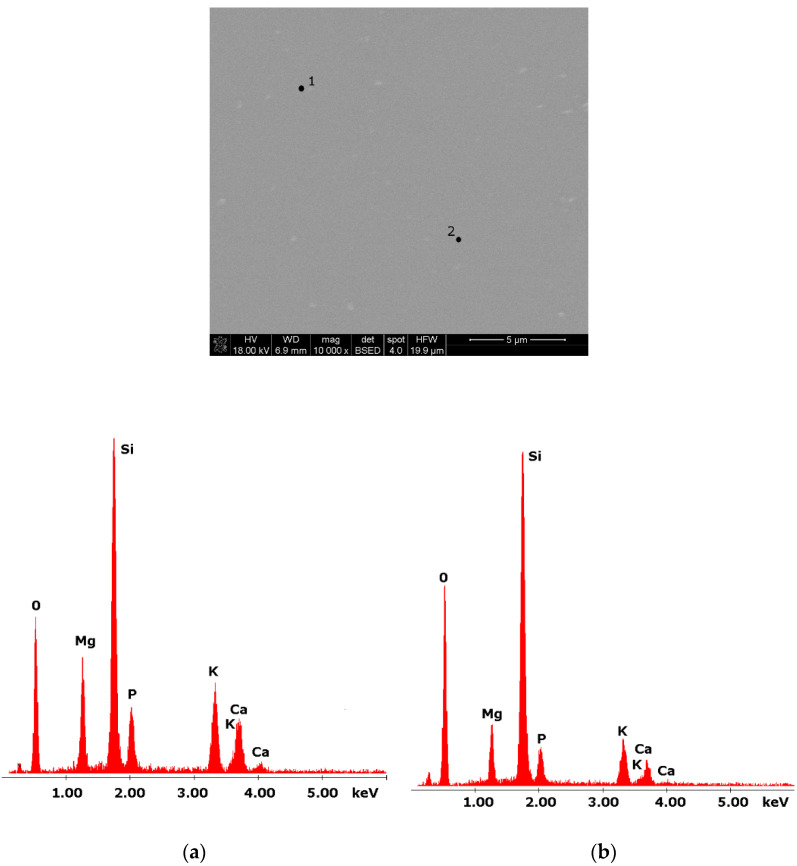
SEM microphotograph of 4B glass. (10,000×): (**a**) EDS analysis of 4B glass (p.1); (**b**) EDS analysis of 4B glass (p.2).

**Figure 2:**
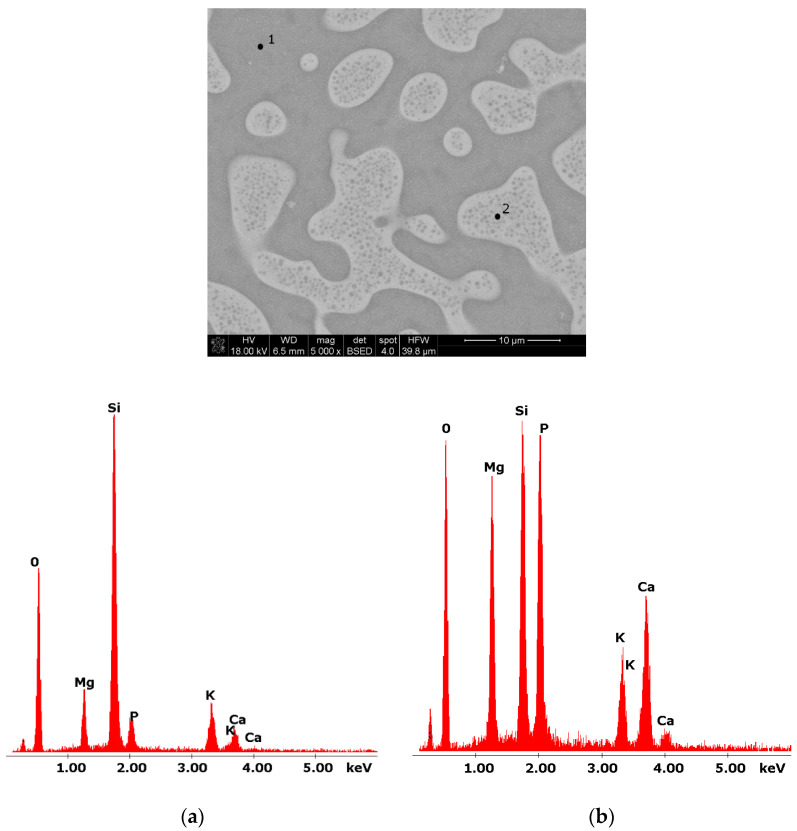
SEM microphotograph of 8B glass. (5000×) (**a**) EDS analysis of 8B glass (p.1); (**b**) EDS analysis of 8B glass (p.2).

**Figure 3:**
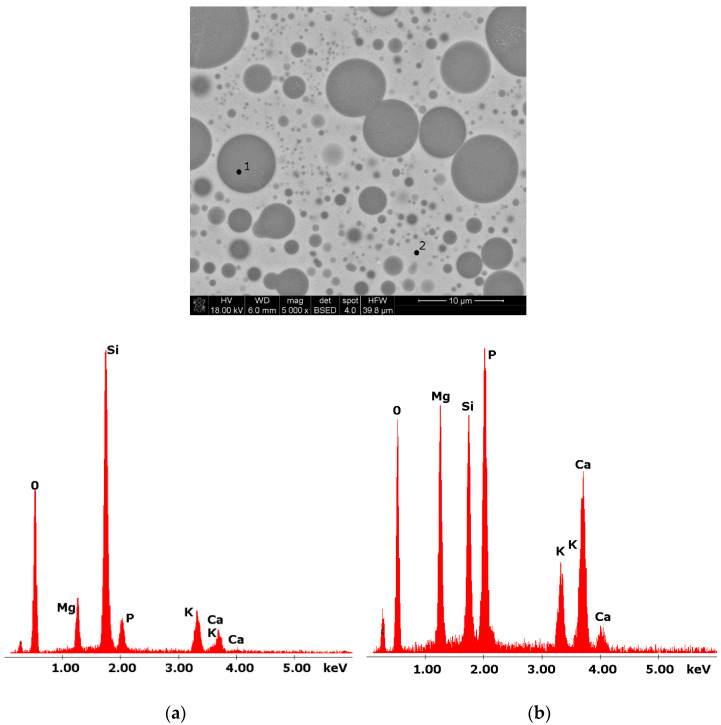
SEM microphotograph of 15B glass. (5000×) (**a**) EDS analysis of 15B glass (p.1); (**b**) EDS analysis of 15B glass (p.2).

Glasses containing B_2_O_3_ in their composition may demonstrate the phenomenon of phase separation. In the case of multi-component silicate–borate–phosphate glasses, it was determined that liquation in the examined system occurs when the B_2_O_3_ content is ≥8 mol.%. A further increase in B_2_O_3_ relative to the composition of the studied glasses, at the expense of MgO and CaO, intensifies this process until the glass is completely opaque. This phenomenon was confirmed by microscopic observations (Figure 1, Figure 2 and Figure 3). The results of SEM-EDS observations showed that both separated phases contained components, i.e., silicon, phosphorus, potassium, magnesium, and calcium, introduced to the glass structure. However, it is worth noting that the semiquantitative EDS analysis showed different amounts of them in different areas of the examined samples. It turned out that one of the phases—later called phosphate—was enriched in P, Ca, and Mg (Figure 2b and Figure 3b), while the other—later called silicate—contained increased amounts of Si and K (Figure 2a and Figure 3a). This observation is crucial for the present study of the crystallization phenomenon, because it allowed the authors to demonstrate that the phenomenon of phase separation has a direct impact on the types of phases formed in the process of crystallization of their amorphous forms.

### 3.2. Glass Stability vs. Phase Separation Phenomenon

**Figure 4:**
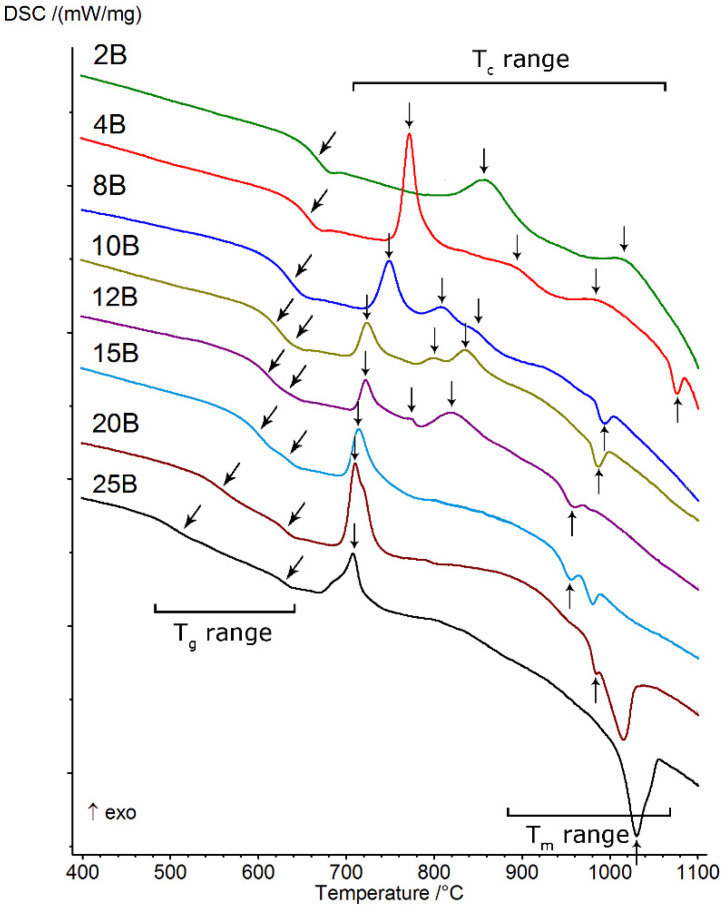
DSC curves of analyzed silicate–borate–phosphate glasses.

The starting point for all of the presented studies was differential scanning calorimetry (DSC), which allowed us to observe transitions typical for the vitreous state, such as the glass transition (T_g_ range), crystallization (T_c_ range), and melting (T_m_ range). All obtained thermal curves are presented in Figure 4, and the values of selected thermal parameters determined from them (Section 2.2) are presented in Table 2. The thermal investigations conducted allowed us to determine the effect of increasing the amount of B_2_O_3_ introduced to the glass composition while diminishing the amounts of MgO and CaO on glass stability, the ability to crystallize, and the course, as well as to identify the types of crystallizing phases. On the basis of the obtained DSC curves, it was found that all of the studied glasses showed the ability to undergo multistage crystallization, the character of which depended on the $\frac{B_{2}O_{3}}{B_{2}O_{3}+\mathrm{MgO}+\mathrm{CaO}}$ ratio (Table 1).

At the lowest value of the above parameter (≤0.042), the crystallization had a two-stage character and occurred in the highest temperature ranges (above 800 °C). As its value increased (≥0.085), the position of the first crystallization effect shifted towards lower temperatures and the character of the crystallization of the studied glasses changed from two-stage to three-stage. From that moment on, in the studied system, we were dealing with three-stage crystallization, which occurred until the value of $\frac{B_{2}O_{3}}{B_{2}O_{3}+\mathrm{MgO}+\mathrm{CaO}}$ exceeded 0.319. Then, the crystallization effect became a single, clearly visible, asymmetrical, exothermic effect.

The parameters determined from thermal curves (K_rg_, ΔT, K_W_, K_H_), which prove the glass stability (GS) of the studied system, are also presented in Table 2. It was found that in the case of glasses that did not exhibit the phase separation phenomenon, for which $\frac{B_{2}O_{3}}{B_{2}O_{3}+\mathrm{MgO}+\mathrm{CaO}}$ ≤ 0.170, the ΔT parameter (as an indicator of GS) displayed gradually decreasing values, which indicates that the ability of the glasses to crystallize was gradually growing at the same time. In the case of liquation glasses ($\frac{B_{2}O_{3}}{B_{2}O_{3}+\mathrm{MgO}+\mathrm{CaO}}$ ≥ 0.212), for which a double effect of glass state transformation was successfully registered on DSC curves, two different tendencies related to their thermal stability were obtained. An analysis of the values indicated in Table 2 showed that, in the case of ΔT_(__1st)_ (ΔT_(1st)_ = T_x_ – (1st)T_g_), the determined values gradually increased, while for ΔT_(2nd__)_ (ΔT_(2nd)_ = T_x_ – (2nd)T_g_), they decreased. The direction of change in parameter ΔT_(2nd__)_ was similar to that found for glasses that did not exhibit phase separation (2B, 4B, and 8B). It seems to the authors of the present study that this two-way behavior may signify that the thermal stability of the analyzed glasses depends, to some extent, on the transformation temperature T_g_ and the character of the separated phases, i.e., the amorphous silicate phase and phosphate phase, formed during the liquation process. This issue is discussed in detail in [24]. The determined for the ΔT parameter may prove that the amorphous phosphate phase, in comparison with the silicate phase, is associated with higher glass stability with respect to devitrification.

Furthermore, it is known that higher values for the K_rg_, K_W_, and K_H_ parameters (Table 2) imply greater resistance to the initiation of the amorphous phase of the crystallization process [2,5,25].

Therefore, the authors of the present study put forward the suggestion that the introduction of increasing amounts of B_2_O_3_ at the expense of MgO and CaO to the composition of glasses from the SiO_2_–B_2_O_3_–P_2_O_5_–K_2_O–MgO–CaO system increases their tendency to undergo crystallization but also contributes to the decreased ease of glass formation.

### 3.3. Crystallization Process

To analyze the phenomenon of crystallization, it was necessary to subject the tested multi-component silicate–borate–phosphate glasses to an isothermal heating process, which was carried out in accordance with the procedure given in Section 2.2. This was related to their significant thermal stability (ΔT), presented in Table 2, and is discussed in more detail in Section 3.2.

The XRD analysis for the obtained devitrificates together with Rietveld refinement showed—despite many hours of heating samples—a relatively small degree of crystallization (Table 3). The amorphous phase represented 55% (for samples heated at higher temperatures, above 800 °C) to 72% (for samples heated in lower temperatures, below 800 °C).

#### 3.3.1. X-ray Diffraction Analysis in the Study of Crystallization Products

**Figure 5:**
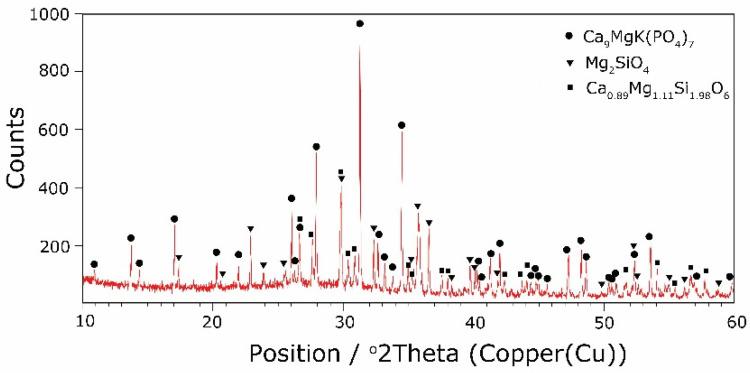
XRD patterns of 2B devitrificate obtained after isothermal heating at 1007 °C (no 2B_1007).

**Figure 6:**
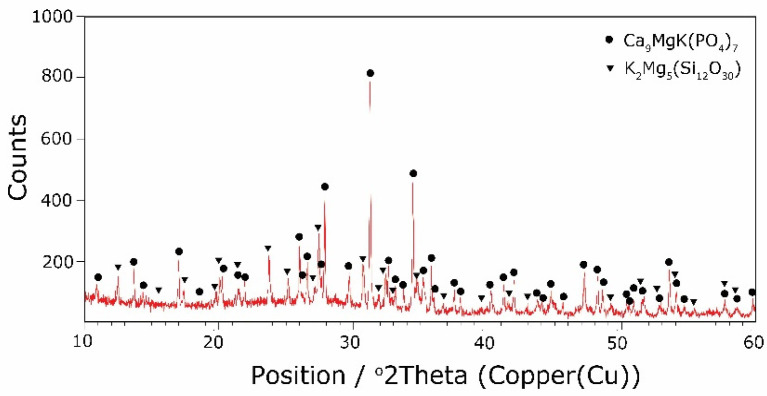
XRD patterns of 10B devitrificate obtained after isothermal heating at 798 °C (no 10B_798).

**Figure 7:**
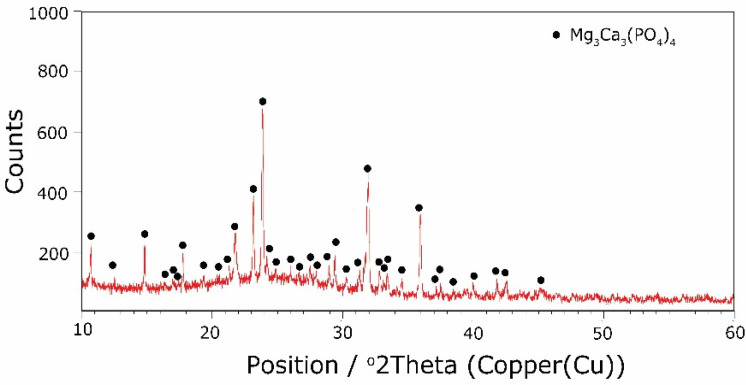
XRD patterns of 25B devitrificate obtained after isothermal heating at 708 °C (no 25B_708).

The types of crystallization phases determined using the XRD method are shown in Table 3. XRD diffractograms of selected devitrificates are presented in Figure 5, Figure 6 and Figure 7.

The obtained results clearly indicate the crystallization of two types of phase: complex phosphates and silicates. First of all, for $\frac{B_{2}O_{3}}{B_{2}O_{3}+\mathrm{MgO}+\mathrm{CaO}}$ < 0.212, a single Ca_9_MgK(PO_4_)_7_-type whitlockite phosphate was crystallized. It is worth noting that this phase was also identified in the sample that was not modified with B_2_O_3_ [24]. However, when the value of $\frac{B_{2}O_{3}}{B_{2}O_{3}+\mathrm{MgO}+\mathrm{CaO}}$ ≥ 0.212 was reached, the phosphate phase Mg_3_Ca_3_(PO_4_)_4_-type stanfieldite was a new product of crystallization in addition to the phosphate mentioned above. At the same time, by performing the Rietveld analysis, we managed to show that with an increase in the $\frac{B_{2}O_{3}}{B_{2}O_{3}+\mathrm{MgO}+\mathrm{CaO}}$ value, the share of the Mg_3_Ca_3_(PO_4_)_4_ phase gradually increased at the expense of the Ca_9_MgK(PO_4_)_7_ phase. Its share increased from about 20% for $\frac{B_{2}O_{3}}{B_{2}O_{3}+\mathrm{MgO}+\mathrm{CaO}}$ = 0.212 to about 72% for $\frac{B_{2}O_{3}}{B_{2}O_{3}+\mathrm{MgO}+\mathrm{CaO}}$ ≥ 0.425 and, finally, up to 100% for $\frac{B_{2}O_{3}}{B_{2}O_{3}+\mathrm{MgO}+\mathrm{CaO}}$ = 0.531. The results of the XRD analysis showed that in higher temperature ranges (above 800 °C), there were more crystallization products and these were more diversified, because apart from the phosphate phases, we also found the presence of silicate phases. Thus, at $\frac{B_{2}O_{3}}{B_{2}O_{3}+\mathrm{MgO}+\mathrm{CaO}}$ ≤ 0.212 in the temperature range from 800 to 910 °C, the presence of the K_2_Mg_5_(Si_12_O_30_)-merrihueite type silicate phase was confirmed next to Ca_9_MgK(PO_4_)_7_. Rietveld’s analysis confirmed that its share, regardless of the $\frac{B_{2}O_{3}}{B_{2}O_{3}+\mathrm{MgO}+\mathrm{CaO}}$-ratio value, did not exceed 16%. In cases where crystallization effects were found at even higher temperatures—exceeding 980 °C—apart from permanently crystallizing phosphate (whitlockite type), silicates with a lower degree of polymerization, i.e., MgSiO_3_-enstatite type, Mg_2_SiO_4_-forsterite type, and CaMgSi_2_O_6_-diopside type were present.

Next, the presence of boron ions in the resulting crystallization products was analyzed. Boron ions were found in both phosphates and silicates. The crystallizing phases were co-created generally in the higher temperature range (800–900 °C), which was at values of 0.085 ≥ $\frac{B_{2}O_{3}}{B_{2}O_{3}+\mathrm{MgO}+\mathrm{CaO}}$ ≥ 0.252. In the case of phosphates, the same non-stichiometric compound, Ca_9.5+0.5x_((PO_4_)_6-x_(BO_3_))(BO_2_)_1-x_O_x_), was always present. In the case of silicates, boron ions were initially present in the silicate K(Bsi_2_O_6_), and then with an increase in the B_2_O_3_ content and decreases in MgO and CaO, its presence was found in the K(Bsi_3_O_8_) silicate.

Considering the above, it is worth mentioning that the results for the type of crystallization phase are in accordance with the results of SEM-EDS analysis (Figure 2 and Figure 3), which are mentioned in point 3.1. It is assumed that the observed phenomenon of liquation in the studied system affects the types of phases formed in the process of crystallization of their amorphous forms. On the basis of the SEM-EDS analysis, we found that the so-called phosphate phase was enriched with phosphorus, calcium, and magnesium (Figure 2b and Figure 3b), while the silicate phase contained increased amounts of silicon and potassium or silicon, magnesium, and potassium (Figure 1a,b, Figure 2a and Figure 3a). Taking this into account and comparing the type of crystallizing phases (Table 3), we can see a significant convergence for the results obtained.

#### 3.3.2. Spectroscopic Methods in the Study of Crystallization Products

**Figure 8:**
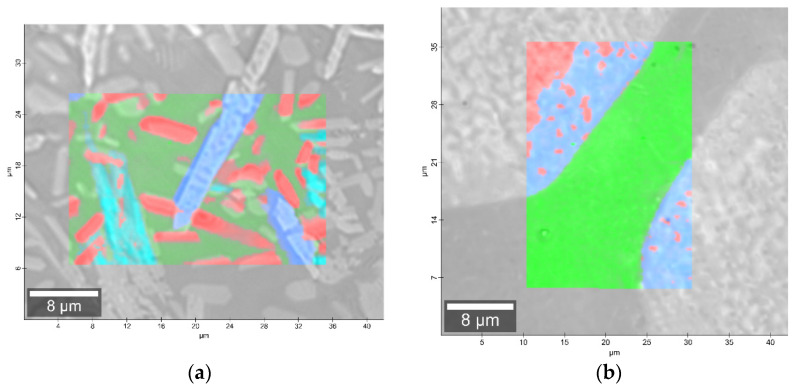
Microscopic images with overlaid Raman images of (**a**) 2B_1007 and (**b**) 20B_710 samples.

**Figure 9:**
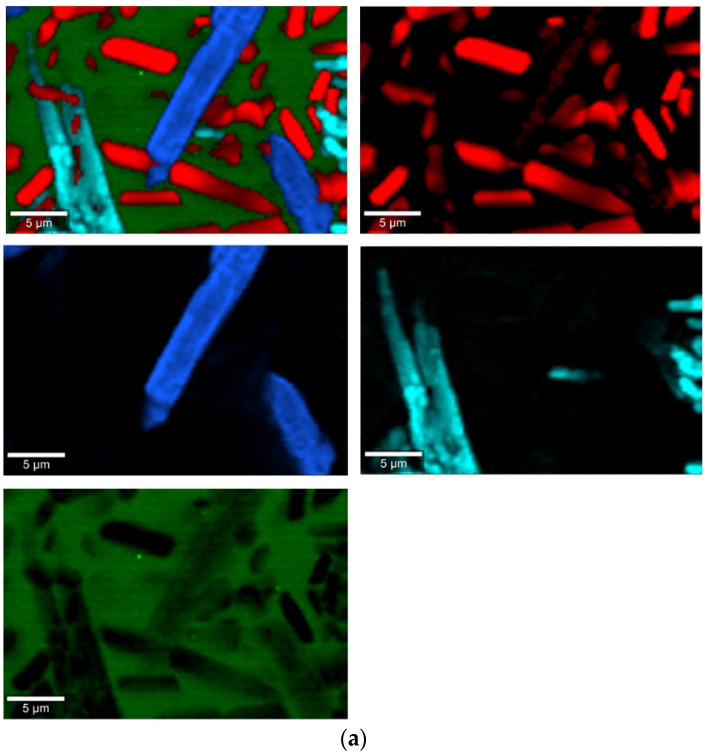

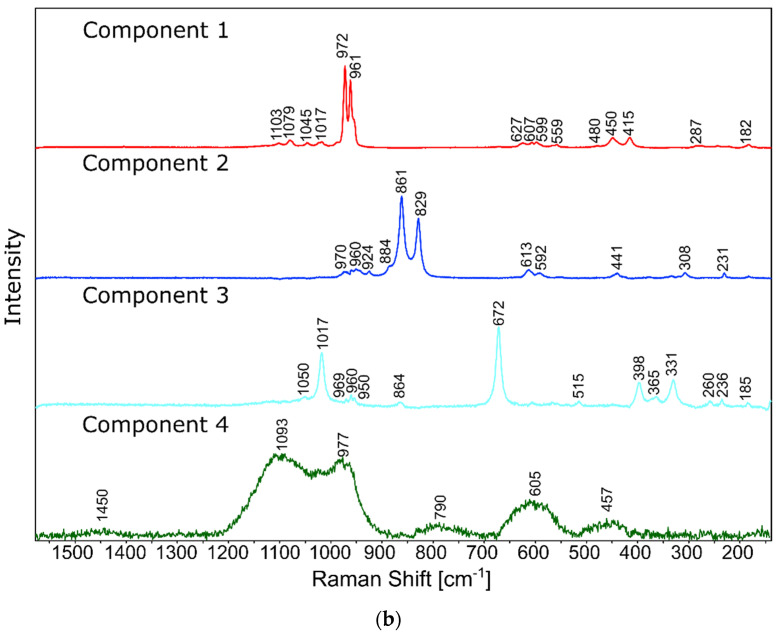
Raman measurements of sample 2B_1007. (**a**) Raman images obtained from the cluster analysis and (**b**) average Raman spectra from corresponding clusters: Component 1—Ca_9_MgK(PO_4_)_7_-whitlockite structure type, Component 2—Mg_2_SiO_4_-forsterite type, Component 3—CaMgSi_2_O_6_-diopside type, Component 4—glass matrix.

**Figure 10:**
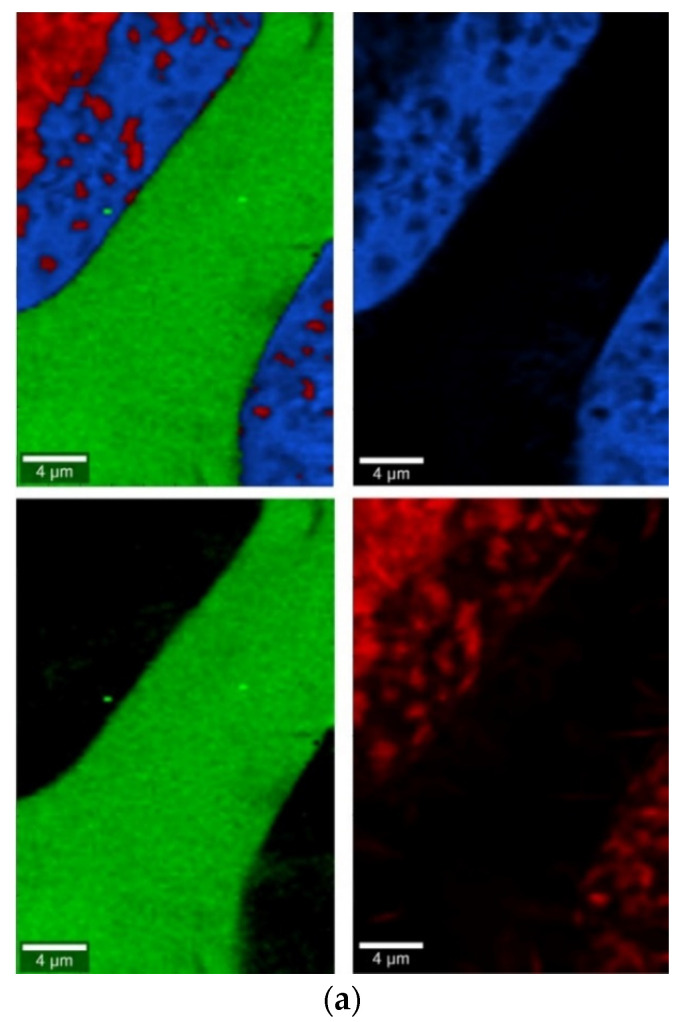

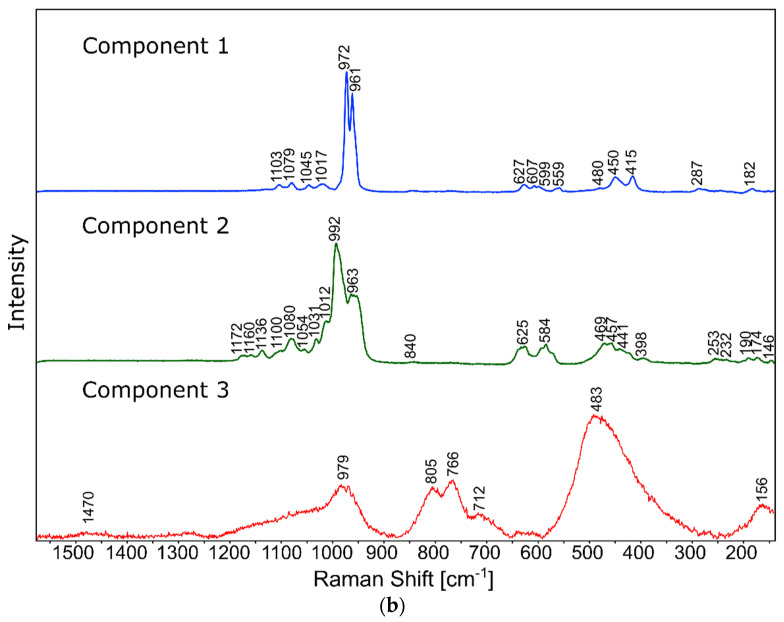
Raman measurements of sample 20B_710. (**a**) Raman images obtained from cluster analysis and (**b**) average Raman spectra from corresponding clusters: Component 1—Ca_9_MgK(PO_4_)_7_-whitlockite structure type, Component 2—Mg_3_Ca_3_(PO_4_)_4_-stanfieldite type, Component 3—glass matrix.

**Figure 11:**
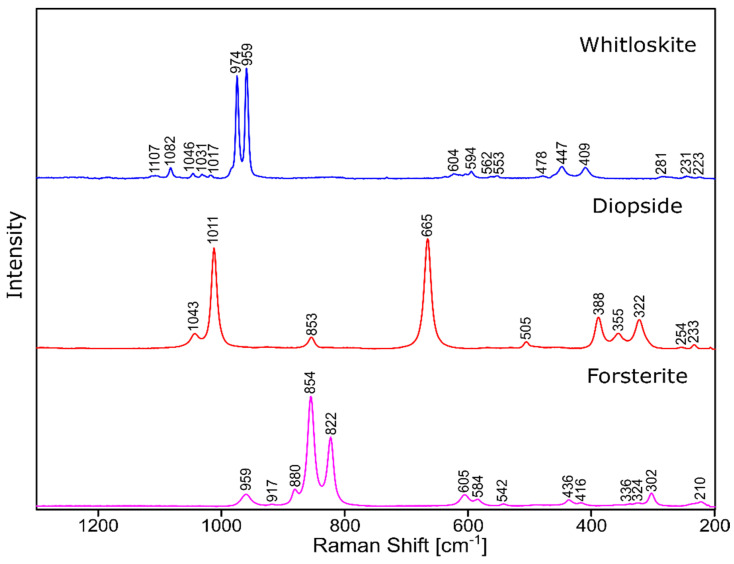
Raman spectra of standard substances.

The phase identification results (XRD analysis) were correlated with RSI (Raman spectroscopy imaging) studies. This method gave us the opportunity to generate detailed chemical images based on the Raman spectra of the sample (Figure 8, Figure 9, Figure 10 and Figure 11). Two devitrificates, nos. 2B_1007 and 20B_710, were selected for the above analysis. They differed significantly in B_2_O_3_ content (Table 1) and phase crystallization type (Table 3). The Raman images obtained are presented in Figure 8, while the collected data from the designated areas are presented in Figure 9 for sample 2B_1007 and Figure 10 for sample 20B_710. Taking into account the information obtained from XRD studies, the identification process for the obtained spectra was related to the Raman spectra of standard substances. These spectra are presented in Figure 11 and they correspond to phosphate Ca_9_MgK(PO_4_)_7_ and two silicates: Mg_2_SiO_4_-forsterite type and CaMgSi_2_O_6_-diopside type.

In the case of the 20B_710 devitrificate, the situation is slightly more complicated. In this case, the glassy matrix (Component 3), marked in Figure 10 with red, was also visible. In this case, similarly to 2B_1007, the observed bands corresponded to the presence of structural units typical for a silicate–phosphate matrix, as described in [19], which also confirmed the presence of boron ions in the glassy matrix. It was demonstrated, among others, the presence of atoms in both triangular (780–800, 147–01470 cm^−1^) and tetraedrical (760–770 cm^−1^) coordination as well as the formation of P–O–B (600–640, 1140–1150 cm^−1^) and B–O–Si (~480 cm^−1^) connections. At the same time, two further components of different structures—Component 1 (blue) and Component 2 (green)—were identified. Based on the Raman spectra obtained (Figure 10), Component 1 (blue) was identified as a phase with a complex phosphate Ca_9_MgK(PO_4_)_7_-whitlockite structure type. Unfortunately, the amount of published data on the Raman spectra of mineral phosphates is still limited, and we could not find a standard Raman spectrum for the phosphate Mg_3_Ca_3_(PO_4_)_4_-stanfieldite type. However, taking into account our detailed and systematic XRD analysis of the series of devitrificates with a gradually changing chemical composition formed on the basis of glasses from the SiO_2_–B_2_O_3_–P_2_O_5_–K_2_O–MgO–CaO system, it seems to be a reasonable proposal that Component’s 2 Raman spectrum (green), presented in Figure 10, comes from the phosphate Mg_3_Ca_3_(PO_4_)_4_-stanfieldite type. Considering phosphate [26,27,28,29], it seems very likely that the green Raman spectra of phosphate oxyanions shows a symmetric stretching mode (ν_1_) at about 992 and 963 cm^−1^, the antisymmetric stretching mode (ν_3_) at about 1012 and 1031 cm^−1^, and the symmetric bending mode (ν_4_) at about 625 and 584 cm^−1^, while bands at about 470 to 398 cm^−1^ could be assigned to the ν_2_ bending mode.

The results presented in Section 3.3.1 and Section 3.3.2 are consistent and complement each other. They confirm that, depending on the temperature, boron ions either create or do not form crystalline phases and then remain in the vitreous matrix. Interestingly, the presence of boron ions in the vitreous matrix was not affected by the amount of B_2_O_3_ introduced into the glass composition but primarily by the crystallization temperature (T_c_)—below or above 800 °C. Only in higher temperature ranges have boron ions been identified in both phosphates and silicates.

### 3.4. Chemical Affinity as a Factor Determining the Type of Crystallizing Phase

An attempt to explain the cause of crystallization of identified crystalline phases turned out to be of particular interest. For this purpose, we used the enthalpy values for the formation of phosphates and silicates from oxides (ΔG) at different temperatures [29], which are presented in Table 4.

It was shown that, in this case, free enthalpy of crystallized compound formation is a factor that determines the type of crystallizing phases. It is known that the phase with the lowest negative ΔG value is the most likely to occur [29]. Thus, the crystallization of phosphates proceeds at the first stage of glass crystallization, while the crystallization of silicates occurs in the higher temperature range. This also applies to the phases containing boron ions. This also applies to the phases containing boron ions, case of which also first crystallize phosphates and then complex silicates (Table 5).

## 4. Conclusions

The main objective of this work was to study the influences of various B_2_O_3_ contents on the structure and crystallization processes of multicomponent glasses from the SiO_2_–B_2_O_3_–P_2_O_5_–K_2_O–MgO–CaO system. Complex multistage crystallization processes induced by the thermal treatment were characterized by SEM-EDS, DSC, XRD, and Raman spectroscopy mapping. It was found that the glass forming ability is limited to the amount of B_2_O_3_ (≤25 mol.%) present, and the phase separation phenomenon occurs with B_2_O_3_ ≥ 8 mol.%. Based on the obtained results, it was possible to link the phenomenon of liquation with the type of crystallizing phase. Simultaneously, it turned out that the increase in the content of B_2_O_3_ introduced while decreasing the contents of MgO and CaO in the silicate–phosphate network diminished the feasibility of undergoing glass formation and increased the ability of the glasses to crystallize. It was also found that the main phases crystallizing in the SiO_2_–B_2_O_3_–P_2_O_5_–K_2_O–CaO–MgO system were complex phosphates and silicates. The sequence of the appearance of crystallization products is governed by the free Gibbs enthalpy of phases formed from oxides; thus, phosphates first crystallize and then silicates. Boron ions appear in both types of crystalline phase but always at higher temperatures (above 800 °C); at lower temperatures, the phenomenon occurs in a vitreous network.

## Figures and Tables

**Table 1:** Nominal compositions of the analyzed glasses from the SiO_2_–B_2_O_3_–P_2_O_5_–K_2_O–MgO–CaO system [mol%].

Name of Sample	SiO_2_	P_2_O_5_	B_2_O_3_	K_2_O	MgO	CaO	$\frac{B_{2}O_{3}}{B_{2}O_{3}+MgO+CaO}$
**2B**	41	6	2	6	27.0	18.0	0.042
**4B**	41	6	4	6	25.8	17.2	0.085
**8B**	41	6	8	6	23.4	15.6	0.170
**10B**	41	6	10	6	22.2	14.8	0.212
**12B**	41	6	12	6	21.0	14.0	0.255
**14B**	41	6	14	6	19.8	13.2	0.297
**15B**	41	6	15	6	19.2	12.8	0.319
**20B**	41	6	20	6	16.2	10.8	0.425
**25B**	41	6	25	6	13.2	8.8	0.531

**Table 2:** Thermal characteristics of the analyzed glasses.

	Glass Transition Range		Crystallization Range						Melting Range	The Glass Stability Parameters (GS)			
			Maximum Crystallization Peaks			Onset of Crystallization Peaks							
**Sample**	T_g_/°C		T_c1_/°C	T_c2_/°C	T_c3_/°C	T_x1_/°C	T_x2_/°C	T_x3_/°C	T_m_/°C	ΔT/°C	K_W_	K_H_	K_rg_
**0B6P** [24]	679		844	896		836			n.o.	157	n.o.	n.o.	n.o.
**2B**	660		858	1007		814	975		n.o.	154	n.o.	n.o.	n.o.
**4B**	651		772	886	988	756	858	952	1068	105	0.113	0.337	0.610
**8B**	631		749	808	847	726	785	829	983	95	0.120	0.370	0.642
**10B**	1st T_g_	626	724			708			988	82	0.099	0.293	0.634
	2nd T_g_	648	724	798	835	708	785	818	988	60	0.077	0.214	0.656
**12B^8^**	1st T_g_	614	722			708			958	94	0.113	0.376	0.641
	2nd T_g_	642	722	775	819	708	n.o.	787	958	66	0.084	0.264	0.670
**15B**	1st T_g_	606	714			699			956	93	0.113	0.362	0.634
	2nd T_g_	634	714			699			956	65	0.084	0.253	0.663
**20B**	1st T_g_	556	710			696			984	140	0.157	0.486	0.565
	2nd T_g_	629	710	720 *		696			984	67	0.082	0.233	0.639
**25B**	1st T_g_	502	708			672			1031	170	0.200	0.474	0.487
	2nd T_g_	628	708	683 *		672			1031	44	0.078	0.123	0.609

* The inflection point of the exothermal effect.

**Table 3:** The kind of crystallizing phases identified in the analyzed devitrificates. Refined evaluation parameters used for the analysis are also reported.

No.	Crystallization Temperature (T_c_) (°C)	Crystalline Phases	Reference Code	Fraction (%)	R_wp_	P_p_	χ^2^
**0B6P** [18]	844	Ca_9_MgK(PO_4_)_7_	n.o.				
	896	Ca_9_MgK(PO_4_)_7_	n.o.				
**2B**	858	Ca_9_MgK(PO_4_)_7_	98-008-5109	77.9	13.66	11.08	1.263
		K_2_Mg_5_(Si_12_O_30_)	98-007-7131	21.1			
	1007	Ca_9_MgK(PO_4_)_7_	98-008-5109	80.5	10.79	9.4	0.854
		Mg_2_SiO_4_	98-020-2370	17.1			
		CaMgSi_2_O_6_	98-001-0224	2.4			
**4B**	772	Ca_9_MgK(PO_4_)_7_	98-008-5109	100	9.4	7.74	1.29
	886	Ca_9_MgK(PO_4_)_7_	98-008-5109	79.9	12.94	10.17	1.223
		K_2_Mg_5_(Si_12_O_30_)	98-007-7131	12.2			
		Ca_9.5+0.5x_((PO_4_)_6-x_(BO_3_))(BO_2_)_1-x_O_x_)	98-006-8336	7.9			
	988	Ca_9_MgK(PO_4_)_7_	98-008-5109	79.9	10.49	8.84	0.72
		MgSiO_3_	98-003-0893	15.8			
		CaMg(Si_2_O_6_)	98-008-9856	3			
		K(BSi_2_O_6_)	98-026-1715	1.3			
**8B**	749	Ca_9_MgK(PO_4_)_7_	98-008-5109	100	10.73	9.39	0.629
	808	Ca_9_MgK(PO_4_)_7_	98-008-5109	85	10.8	9.15	0.681
		K_2_Mg_5_(Si_12_O_30_)	98-007-7131	15			
	847	Ca_9_MgK(PO_4_)_7_	98-008-5109	81.8	10.1	8.4	0.747
		K_2_Mg_5_(Si_12_O_30_)	98-007-7131	7.5			
		Ca_9.5+0.5x_((PO_4_)_6-x_(BO_3_))(BO_2_)_1-x_O_x_)	98-006-8336	8.4			
		K(BSi_2_O_6_)	98-026-1715	2.3			
**10B**	724	Ca_9_MgK(PO_4_)_7_	98-008-5109	75.2	10.98	9.48	0.634
		Mg_3_Ca_3_(PO_4_)_4_	98-002-3642	24.8			
	798	Ca_9_MgK(PO_4_)_7_	98-008-5109	84	10.77	9.12	0.606
		K_2_Mg_5_(Si_12_O_30_)	98-007-7131	16			
	835	Ca_9_MgK(PO_4_)_7_	98-008-5109	81.9	10.91	8.96	0.718
		K_2_Mg_5_(Si_12_O_30_)	98-007-2717	7.9			
		Ca_9.5+0.5x_((PO_4_)_6-x_(BO_3_))(BO_2_)_1-x_O_x_)	98-006-8336	6.3			
		K(Bsi_2_O_6_)	98-026-1715	4			
**12B**	722	Ca_9_MgK(PO_4_)_7_	98-008-5109	80.6	10.64	9.31	0.581
		Mg_3_Ca_3_(PO_4_)_4_	98-002-3642	19.4			
	819	Ca_9_MgK(PO_4_)_7_	98-008-5109	45.1	11.87	10.22	0.807
		Mg_3_Ca_3_(PO_4_)_4_	98-002-3642	41.9			
		K(Bsi_3_O_8_)	98-006-9445	8.2			
		Ca_9.5+0.5x_((PO_4_)_6-x_(BO_3_))(BO_2_)_1-x_O_x_)	98-006-8336	4.7			
**15B**	714	Ca_9_MgK(PO_4_)_7_	98-008-5109	37.9	11.19	9.59	0.643
		Mg_3_Ca_3_(PO_4_)_4_	98-002-3642	62.1			
**20B**	710	Ca_9_MgK(PO_4_)_7_	98-008-5109	8	14.04	11.9	0.931
		Mg_3_Ca_3_(PO_4_)_4_	98-002-3642	72			
**25B**	708	Mg_3_Ca_3_(PO_4_)_4_	98-002-3642	100	14.63	12.27	0.938

**Table 4:** Values of ΔG for the formation of silicates and phosphates crystallizing in the analyzed glasses.

Compounds		ΔG [kJ/mol]		
		900 K	1000 K	1100 K
Ca_9_MgK(PO_4_)_7_	9CaO·MgO·0.5K_2_O·3.5P_2_O_5_	–18,846.6	–19,196.9	–19,563.9
K_2_Mg_5_(Si_12_O_30_)	K_2_O·5 MgO·12 SiO_2_	–15,336.6	–15,534.0	–15,743.5
Mg_3_Ca_3_(PO_4_)_4_	3MgO·3CaO·2P_2_O_5_	–10,716.1	–10,909.2	–11,111.5
CaMg(Si_2_O_6_)	CaO·MgO·2SiO_2_	–3271.4	–3311.9	–3354.8
Mg_2_SiO_4_	2MgO·SiO_2_	–2255.3	–2282.7	–2311.8
MgSiO_3_	MgO·SiO_2_	–1612.6	–1632.0	–1652.7

**Table 5:** Values of ΔG for the formation of silicates and phosphates containing boron crystallizing in the analyzed glasses.

Compounds		ΔG [kJ/mol]		
		900 K	1000 K	1100 K
Ca_9.93_(P_5.84_B_0.16_O_24_)B_0.67_O_1.79_	9.93CaO·2.92 P_2_O_5_·0.415B_2_O_3_	–17,217.4	–17,526.0	–17,849.5
K(BSi_2_O_6_)	0.5K_2_O·0.5B_2_O_3_·2SiO_2_	–2861.4	–2904.3	–2949.9
K(BSi_3_O_8_)	0.5K_2_O·0.5B_2_O_3_·3SiO_2_	–3831.3	–3885.7	–3943.3

## Data Availability

Data available on request due to privacy restrictions. The data presented in this study are available on request from the corresponding author.

## Sample Availability

Samples of the compounds are not available from the authors.
